# Mutational signatures in hematological malignancies

**DOI:** 10.31744/einstein_journal/2026RW1961

**Published:** 2026-02-02

**Authors:** Felipe de Almeida Sartori, João Vitor Paes Pontes, Paulo Vidal Campregher, Thomas LaFramboise

**Affiliations:** 1 Case Western Reserve University Cleveland Ohio United States Case Western Reserve University, Cleveland, Ohio, United States.; 2 Hospital Israelita Albert Einstein São Paulo SP Brazil Hospital Israelita Albert Einstein, São Paulo, SP, Brazil.; 3 Genesis Genomics São Paulo SP Brazil Genesis Genomics, São Paulo, SP, Brazil.

**Keywords:** Mutational signatures, Hematological malignancy

## Abstract

The set of somatic mutations present in a human tumor is a record of one or more mutational processes, each of which leaves distinct "signature" of mutation types. Mutation types can be classified in various ways, the most straightforward being the base change induced by a single-base substitution (*e.g*., C>A, T>G, etc.). The advent of high-throughput DNA sequencing has facilitated the comprehensive, genome-wide assessment of mutation types in human tumors. This has spurred the development of methodology to tease apart the relative contribution of each mutational process by decomposing the set of all mutations into individual signatures. Many mutational signatures have known etiologies. Therefore, mutational signature inference can shed light on the causes of cancer and inform patient treatment. To date, most studies in this area have been performed on solid tumors; consequently, the application of existing methods to hematological cancers has yielded limited results. In this review, we provide an overview of the history and methodology behind mutational signature inference. Here, we present the challenges inherent in its application to hematological cancers and survey the work performed thus far. We highlight how recent research analyzing mutational signatures in normal blood cells can elucidate the beginning of a continuum of mutational processes, from normal hematopoiesis through mature hematological malignancy. Accurate characterization of mutational signatures in cancer development may aid in clinical diagnosis, prognosis, and treatment decisions.

## INTRODUCTION

Since the advent of high-throughput sequencing technologies, our understanding of the genetic underpinnings of human diseases has dramatically expanded.^(1)^ Among the conditions most impacted by these advances is cancer, which is fundamentally driven by the accumulation of somatic mutations.^(2)^ The ability to decode entire genomes has propelled research, providing unprecedented insights into the molecular basis of tumorigenesis and opening avenues for personalized medicine.^(3)^

A particularly intriguing discovery, dating back to the early 2000s, was the observation that mutations tend to occur in distinct patterns that are often associated with specific exposures. For instance, tumors arising in sun-exposed skin commonly exhibit C>T and CC>TT transitions, reflecting DNA damage induced by ultraviolet (UV) light.^(4)^ These recurring mutation patterns, now known as mutational signatures, suggest that a tumor's mutational landscape carries a historical imprint of the biological processes and external agents that shaped its evolution.^(3,5,6)^

Although groundbreaking, early studies of mutational patterns have faced several limitations.^(5–7)^ Most focused exclusively on driver genes, which are under selective pressure, potentially obscuring the full spectrum of mutations. Moreover, these studies typically analyzed a limited number of mutations per sample and lacked robust computational tools to disentangle overlapping mutational processes. Consequently, these studies have only provided a partial view of the mutational processes operating in cancer genomes.

With the rise of whole-genome sequencing (WGS), it has become possible to catalog nearly all somatic mutations in a given tumor.^(3)^ This technological leap facilitated a more comprehensive approach to mutational analysis. A pioneering study by Nik-Zainal et al. applied a mathematical framework based on non-negative matrix factorization (NMF) to identify mutational signatures from large-scale sequencing data of 21 breast cancer genomes.^(6)^ Subsequent work by Alexandrov et al. refined and publicly released the model.^(7)^ By incorporating all mutations within each tumor sample and analyzing patterns across multiple samples, this method revealed distinct mutational signatures, some of which could be linked to specific biological mechanisms or exposures - such as UV light.

Initially, these efforts focused on single base substitutions (SBSs), categorized using a 96-mutation framework that considers the trinucleotide context of each mutation (the mutated nucleotide plus its immediate 5’ and 3’ neighbors).^(5)^ This allowed researchers to decompose complex mutational spectra into interpretable components. Over time, the scope of analysis broadened to include additional mutation types such as doublet base substitutions,^(8)^ small insertions and deletions,^(8)^ copy number alterations,^(9, 10)^ structural variants,^(11)^ and even RNA-based mutations.^(12)^

As the field matured, the catalogue of known mutational signatures expanded significantly. New computational tools tailored to different data types and research goals have emerged, enabling more precise and personalized analyses.^(13–23)^ Recent discoveries have further elucidated the links between certain signatures and their underlying etiologies.^(8)^ To support ongoing research and clinical use, the COSMIC (Catalogue of Somatic Mutations in Cancer) database^(24)^ now maintains a curated and evolving collection of mutational signatures, currently in version 3.4, serving as a key resource for the scientific and medical communities.

This review provides an overview of the development and current state of mutational signature research. We describe the methodological advances that have enabled the creation of analytical tools for signature extraction, compare existing approaches, and explore the applications of mutational signatures in both research and clinical contexts. Finally, we discuss the current state of mutational signature research in hematological malignancies and its potential in this domain.

## EXISTING TOOLS TO EXTRACT MUTATIONAL SIGNATURES

A mutational signature, assumed to be caused by a single biological process or exogenous exposure, is a pattern of mutation types specified by the percentage of each mutation type. The simplest mutation types are SBSs. Considering the redundancy of the two complementary DNA strands, there are six possible substitutions: C>A, C>G, C>T, T>A, T>C, and T>G. As flanking nucleotides can also influence mutagenic processes, SBS signatures also consider the bases on the 3′ and 5′ sides of the mutation site, therefore yielding 4 × 6 × 4 = 96 possible types of SBS mutations. Since the first tool was reported in 2012, tools for mutational signature extraction have undergone significant developments. In addition to refinements of NMF, alternative mathematical approaches have been introduced.^(16,18–21)^

The original tool developed by Alexandrov et al., SigProfiler, has since been replaced by SigProfilerExtractor^(15)^ and SigProfilerAssignment,^(13)^ both of which were developed by the same laboratory but designed for distinct purposes. SigProfilerExtractor is intended for *de novo* extraction, whereas SigProfilerAssignment focused on signature fitting.

*De novo* extraction refers to the process of uncovering mutational signatures directly from a dataset (typically WGS data), without using previously-reported signatures.^(25)^ This approach allows researchers to reveal both novel and known signatures. Computational comparisons, such as cosine similarity, are commonly employed to determine whether extracted signatures correspond to known signatures.

In contrast, the fitting strategy uses a predefined reference signatures, typically in the COSMIC database, to estimate the contribution of each known signature to the mutational profiles of individual samples. The COSMIC database^(24)^ catalogs well over 100 human mutational signatures. Therefore, it is feasible to determine the composition of mutational signatures acting on a tumor by mathematically inferring the proportion of each known signature using the tumor's counts of each type of mutation as input data.

In addition to the contributions of Alexandrov et al., several other research groups have developed tools with various features and purposes. The main differences among these tools include the type of extraction (*de novo versus* fitting), input format (usually VCF files or mutational count matrices), mathematical approach, and intended application (e.g., some are optimized for specific cancers, such as mmsig for hematologic malignancies^(22)^).

Other tools available for signature extraction include PLDA,^(19)^ MCSM,^(17)^ SigTracer,^(20)^ MutationalPatterns,^(18)^ YAPSA (Yet Another Package for Signature Analysis),^(14)^ mmsig,^(22)^ MUSE-XAE^(21)^ and MuSiCal.^(16)^ A comparative description of these tools and their characteristics is provided in table 1.

**Table 1:** Description of a selection of tools designed for mutational signature extraction

Tool name	Data types	Mathematical approach	Extraction type	Special trait
MCSM	WGS	MMM	Fitting	Evaluate strand or genomic region bias on genomic processes
Mix	Gene panel	MMM	Fitting	Use with clinical sequencing
mmsig	WGS	EM algortihm	Fitting	Built for hematologic malignancies
MUSE-XAE	WGS	Nonlinear encoder and linear decoder	*De novo* and fitting	-
MuSiCal	WES, WGS (preferably)	mvNMF and likelihood-based sparse NNLS	*De novo* and fitting	-
MutationalPatterns	WGS	NMF and NNLS	*De novo* and Fitting	Understand regional mutation spectra and strand asymmetry
Palimpsest	WES, WGS	NMF	*De novo* and Fitting	-
PLDA	WGS	PLDA	Fitting	Allowing the use of the same reference for all cancer types
SATS	Gene panel	pNMF and EM	*De novo* and fitting	Use with clinical sequencing
SigMA	Gene panel	NMF and NNLS	*De novo* and fitting	Mutational signature associated with HR deficiency
SigProfilerAssignment	WES, WGS (preferably)	NNLS	Fitting	-
SigProfilerExtractor	WES, WGS (preferably)	NMF and NNLS	*De novo* and fitting	-
SigTracer	WGS	Hierarchical Bayesian approach	Fitting	Analyze intra-tumor heterogeneity
YAPSA	WES, WGS (preferably)	NNLS	Fitting	-

EM: expectation-maximization; HR: homologous recombination; MCSM: mutation-level covariate signature model; MMM: multinomial mixture model; mvNMF: minimum-volume NMF; NMF: nonnegative matrix factorization; NNLS: nonnegative least squares; PLDA: parallelized latent Dirichlet allocation; pNMF: poisson NMF; WES: whole-exome sequencing; WGS: whole-genome sequencing; YAPSA: yet another package for signature analysis.

Given the proliferation of tools and methods, two essential questions emerge: (i) What is the optimal strategy for extracting mutational signatures? (ii) Which tools are most effective? The literature reflects substantial variation in approaches, often leading to different results, even when applied to similar datasets.^(16)^ Two key studies have provided insight into these questions. Maura et al. proposed a structured workflow for signature extraction^(25)^ with a focus on hematological malignancies, while Medo et al. conducted a comparative evaluation of different tools.^(26)^

According to Maura et al., three main challenges complicate signature extraction:

- Nonuniqueness: Different combinations of signatures may explain the same mutational profile, especially for flat signatures that lack distinct peaks across trinucleotide contexts.
- Localized processes: Some mutational processes are region-specific and may be diluted when analyzing the genome as a whole.
- Signature bleeding: Signatures are erroneously assigned to samples in which they are not biologically present. This often results from pipelines that assume uniform exposure across heterogeneous cohorts.

To address these challenges, Maura et al. recommended a three-step workflow.

1. Begin with *de novo* extraction to identify cohort-specific signatures,
2. Map these to known references (*e.g.*, COSMIC) using similarity metrics,
3. Perform fitting using the identified signatures as a reference to quantify their contribution in each sample.

They also suggested applying this workflow to specific genomic regions as a strategy to address the issue of localized mutational processes. By focusing on regions known to be differentially affected in the cohort of interest, it becomes possible to detect signatures that would be missed in a whole-genome approach.

Medo et al.^(26)^ found that tool performance varies depending on several factors, such as mutation burden per sample, type of downstream analysis, and cancer type. Their results indicated that while SigProfilerSingleSample (now discontinued) performed well in low-mutation samples, SigProfilerAssignment and MuSiCal were better-suited for high-mutation datasets.

Additionally, significant progress is being made in extracting mutational signatures from sequencing methods other than WGS, such as whole-exome sequencing (WES)^(15)^ and targeted panels.^(27–30)^ Given the clinical predominance of WES and targeted approaches, these developments are critical for translating mutational signature research into practical healthcare applications. Moreover, the increased depth of coverage achievable by these methods can uncover low-frequency mutations that may be missed by WGS.^(31)^ Tools capable of performing signature extraction in such contexts are listed in table 1, along with those designed for WGS data.

## MATHEMATICS UNDERLYING THE MUTATIONAL SIGNATURE ANALYSIS

In their simplest form, algorithms to infer molecular signatures *de novo* take as input a 96 × *M* matrix *A*, whose columns represent the *M* tumors sequenced, and whose rows represent all possible SBS mutation types. In its row *i*, column *j* entry, the input matrix *A* has the number of mutations of type *i* in tumor *j*.

The algorithm has two goals:

1. Infer the composition (*i.e.*, prevalence of each mutation type) of each individual mutational signature present in the sample set.
2. Infer what proportion each mutational signature contributes to each tumor's molecular landscape.

The main mathematical concept underlying this algorithm is NMF.^(32)^ Briefly (for technical details, see ^(7)^), the aim is to represent *A* as the product of two matrices *W* and *H*, that is *A* ≈ *W* * *H* where * denotes matrix multiplication. *W* is a 96 × *k* matrix whose rows represent the types of mutations and whose columns represent *k* distinct mutational signatures. *W* addresses Goal 1 above, as its columns give the prevalence of each mutational type in each of the *k* signatures. *H* is a *k* × *M* matrix that addresses Goal 2 by providing the relative contribution of each signature to an individual tumor in each column. The factorization *A* ≈ *W* * *H* (i.e. determining the matrices *W* and *H*) is typically performed using an iterative procedure that aims to find the entries for *W* and *H* that minimize the difference between *A* and *W* * *H*, computed as ||*A* - *W* * *H*||*_F_*, where ||*X||_F_* represents the Frobenius norm of *X*, defined as


$${\left\|X\right\|}_{F}=\sqrt{\sum_{i=1}^{N}\sum_{j=1}^{M}x_{ij}^{2}}$$

for any *N* x *M* matrix *X* with *i*, *j* entry *x_ij_*.

Note that the factorization *A* = *W* × *H* as described here, assumes knowledge of the number *k* of distinct mutational signatures present in the set of tumors. In practice, *k* is unknown *a priori*. To determine the optimal value for *k*, the minimization procedure described above is typically run multiple times for each of several values of *k*. Each value of *k* is assessed for reproducibility and stability across the multiple runs. Also considered is the error between each tumor's actual mutation pattern, as catalogued in matrix *A*, and that approximated by *W* × *H*. The value of *k* that is most reproducible, stable, and best mitigates the error rate is selected as the optimal value for *k*.

The methodology for extracting signatures using mutations other than SBS (DBS, ID, CN, SV, and RNA-based) is similar. There are also multiple alternative variants of the *de novo* extraction procedure; however, most are conceptually similar to the approach described here.

Unlike *de novo* extraction, fitting uses known and specific mutational signatures. Suppose there are *n* known SBS mutational signatures under consideration. They can be represented by a 96 × *n* matrix *S*, the columns of which provide the proportion of each mutation type in the corresponding signature. The counts of different single-base substitutions in a tumor may be represented as vector *v* with 96 entries, each entry corresponding to the number of mutations of one of the 96 types described above. Determining the optimal assignment of relative mutational signature contributions to the tumor mathematically corresponds to determining the vector *a* of length *n* that minimizes the difference between *v* and *S* × *a*. Here, the 96 entries in *a* give the estimated relative contributions of each mutational signature to the mutational spectrum of the tumor. The difference between *v* and S*a is computed as ||*v* - *S* × *a*||, where ||*x*|| denotes the length of vector *x*, defined as


$$\left\|x\right\|=\sqrt{\sum_{i=1}^{96}x_{i}^{2}}$$

for a vector of length 96.

The naïve determination of the optimal value of *a* assumes that all *n* mutational signatures may be present, leading to overfitting. One approach to avoid overfitting is to iteratively remove and add signatures and determine how the difference ||*v* - *S* × *a*|| is affected. The specific steps of this "forward stepwise" algorithm are outside the scope of this review, but details of one implementation may be found in Díaz-Gay et al.^(13)^ In the end, some subset of the *n* mutational signatures are omitted from consideration, which results in a more reasonable number of inferred signature contributions.

## CLINICAL RELEVANCE OF MUTATIONAL SIGNATURE INFERENCE

Inferring mutational signature composition from tumor DNA sequence can reveal the estimated number of mutations caused by each contributing signature. Aside from basic scientific insights, what more practical value might mutational signature inference have, particularly in the context of myeloid malignancies?

Some of the mutational signatures curated by COSMIC have known or suspected etiology. The initial and most well-established of these are signatures that were found in the first whole cancer genomes to be sequenced. These signatures are associated with smoking (particularly in lung cancer)^(33)^ and UV light (particularly in skin cancer).^(34)^ The primary cause of a tumor with mutation patterns dominated by one of these signatures would presumably be the corresponding exogenous exposure. Subsequently, several other exogenous causes of mutational signatures were identified. Treatments (for cancer and otherwise) associated with specific mutational signatures include temozolomide, platinum chemotherapy, azathioprine, thiopurine, duocarmycin, and melphalan.^(35)^ Some of these signatures are particularly pronounced in post-treatment tumors with underlying mismatch repair (MMR) deficiency. Therefore, treatment of MMR-deficient patients may actually induce mutations that drive relapse or resistance to the treatment itself. For instance, acute lymphoid leukemia (ALL) patients treated with thiopurine chemotherapy were shown to acquire known relapse-associated mutations in the genes *NR3C1*, *TP53*, and *NT5C2.*^(36)^

MMR-deficiency signatures are among the many that arise endogenously, whether from specific gene mutations or by other means. Endogenously-derived signature etiologies include 5-methylcytosine deaminase and apolipoprotein B editing complex (APOBEC) activity, polymerase eta errors, base excision repair deficiency, reactive oxygen species (ROS) damage, and homologous recombination (HR) deficiency, among others. Clustering based on some of these mutational signatures in multiple myeloma yielded groups with prognostic value.^(37)^ Therefore, specific compositions of mutational signatures have the potential to inform clinical treatment and surveillance decisions.

It should also be noted that the presence of certain mutational signatures may indicate greater sensitivity to specific cancer therapeutics. The most well-established example is that the presence of HR-deficiency signatures is closely associated with sensitivity to both platinum-based chemotherapy and poly(ADP)-ribose polymerase (PARP) inhibitors in breast cancer. Other examples with potential clinical applications include APOBEC signatures and their association with sensitivity to ataxia telangiectasia and Rad3-related kinase (ATR) inhibition,^(38)^ ROS damage signatures associated with *MYCN* amplification^(39)^ and potentially with sensitivity to electron transport chain complex I inhibitors.^(40)^ The potential link between ROS damage and therapeutic sensitivity may be particularly relevant in acute myeloid leukemia (AML), as McLeod et al. found a ROS damage mutational signature in a substantial proportion of pediatric and adult AML samples.^(41)^

## ANALYTIC APPROACHES FOR MUTATIONAL SIGNATURE INFERENCE IN HEMATOLOGICAL MALIGNANCIES

**Figure 1:**
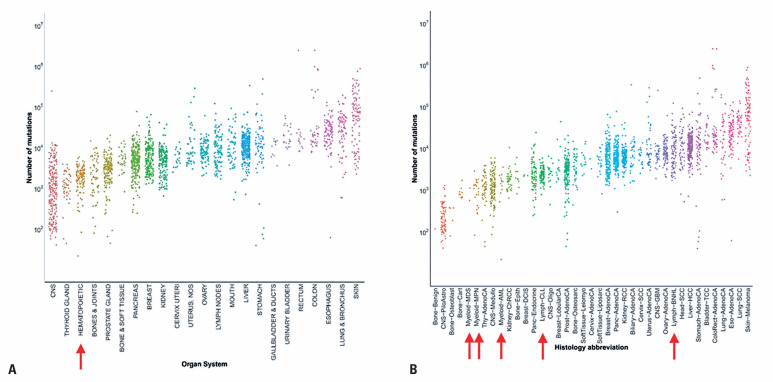
Somatic mutation rates tend to be lower in hematological malignancies. (A) Total numbers of mutations for each tumor in the Pan-Cancer Analysis of Whole Genomes, grouped by tissue type. Hematological tumors are indicated with red arrow. (B) Same as panel (A), but here, tumors are grouped by specific cancer type. Red arrows indicate hematological cancers. Note that all hematological cancers are among the less frequently mutated types, save B-cell non-Hodgkin lymphoma (BNHL)

The vast majority of work in cancer mutational signature analysis have been conducted in solid tumors.^(6,42–46)^ Indeed, there are inherent challenges in the accurate calling of mutational signatures in hematological cancers. One issue is that the mutation rate tends to be much lower than that in solid tumors (Figure 1). This is particularly true of myeloid malignancies. For example, AML typically has some 100-fold fewer mutations than lung cancer and melanoma. Even calling somatic mutations is more difficult in blood cancers than in solid tumors.^(47)^ To distinguish between somatic and germline variants in tumor DNA, researchers often rely on matched normal DNA from the same patient. Blood is a convenient source of normal DNA in patients with solid malignancies; however, it is unsuitable for hematological malignancies. Buccal swabs, fingernails, hair, saliva, and skin can serve as alternative sources of normal DNA. However, all of these sources have drawbacks, such as inadequate amounts of DNA or contamination with malignant blood cells. Some researchers culture skin fibroblasts; however, this is not a scalable solution.^(48)^

Another challenge is that *de novo* signature extraction requires a large patient cohort.^(25)^ Hematological cancers are generally much rarer than many solid tumor types, which limits the number of available whole-genome sequences. In existing literature, the dominant signatures in blood cancers are "clock-like" signatures that are closely linked to aging.^(22)^ As a result, lower-abundance signatures can be difficult to detect accurately.

Despite these challenges, several groups have developed strategies to identify mutational signatures in hematological malignancies using standard tools. Researchers at the Memorial Sloan Kettering Cancer Center developed a platform, termed MSK-IMPACT Heme^(47)^ which sequences 400 targeted genes that are known to play roles in hematological cancers. The use of a targeted panel greatly reduces costs compared to whole-genome sequencing and makes the sequencing of matched normal DNA economically feasible. In their study, the authors used either nail clippings or saliva as germline DNA sources. Owing to the limited number of mutations that can be detected by targeted sequencing, SigProfiler was applied to only the 11% of tumors with the highest mutational burden, all but two of which were lymphoid malignancies. In addition to age-related signatures, a variety of other signatures were detected, largely corresponding to the type of treatment that the patient received prior to sequencing.

Diamond et al.^(35)^ obtained similar results using whole-genome and whole-exome sequencing of myeloid malignancies, also applying SigProfiler. They exclusively found age-related signatures in treatment-naïve AML and patients undergoing radiation, but found treatment-related signatures in patients undergoing either high-dose melphalan- or platinum-containing therapies. In both cases, the study used standard methods to infer mutational signatures without tailoring them to hematological malignancies.

Hoang et al.^(37)^ used the Palimpsest method,^(49)^ primarily developed for solid tumors, to infer mutational signatures from whole-genome and whole-exome sequencing of multiple myeloma samples. The authors identified multiple "flat" signatures, including one associated with homologous recombination repair deficiency, which was subsequently criticized as being a false positive finding.^(22)^ As mentioned previously, flat signatures are difficult to distinguish from one another.

In response to some of these challenges, Rustad et al.^(22)^ developed a method called *mmsig*, which was specifically designed for hematological malignancies. The authors demonstrated its efficacy on 82 multiple myeloid samples from the CoMMpass project,^(50)^ 142 chronic lymphocytic leukemia (CLL) patients from the CLL Genome Project,^(51)^ and AML patients from TCGA.^(52)^ The approach uses a dynamic error-suppression procedure rather than relying on a hard percentage cutoff for signature contributions. They were able to show the ability to accurately infer mutational signatures, even from low numbers of mutations.

## MUTATIONAL SIGNATURES IN HEMATOLOGICAL MALIGNANCY AND THEIR PRECURSORS: A SURVEY OF DISCOVERIES THUS FAR

All hematological malignancies arise in cells with normal blood cell progenitors. Deep sequencing of hematopoietic stem cells (HSCs) has revealed distinct mutational signatures, even at very early stages of life, including in cord blood.^(53)^ Some mature malignancies pass through precursor stages during which cells acquire additional mutations that confer a proliferative advantage.

In the myeloid realm, one stepwise path is from normal HSCs to clonal hematopoiesis of indeterminate potential (CHIP) to clonal cytopenia of unknown significance (CCUS) to myelodysplastic syndrome (MDS) to AML. Along this continuum, mutational signatures have only been characterized in normal HSCs, CHIP, and AML. In individuals without mutagenic exposures such as smoking and chemotherapy, mutational signatures in all these entities tend to be dominated by the clock-like signatures SBS1 and SBS5. (Note that here we use the nomenclature adopted from the COSMIC database https://cancer.sanger.ac.uk/signatures/). SBS1 is caused by spontaneous deamination of methylated cytosines into thymines and is very closely tied to the cell cycle. The cause of the SBS5 signature is unknown, although it is strongly correlated with age. SBS32 has also been reported to be common in normal HSCs and AML.^(53)^ However, its association with azathioprine treatment^(54)^ raises questions regarding its validity. The observation of a dominant and consistent mutational pattern in normal HSCs led some researchers to define the overall pattern as its own signature, termed SBS-HSC.^(31,35)^ Studies querying mutational signatures in CHIP have, unsurprisingly, revealed SBS-HSC or its components as dominant for the majority of individuals.^(55,56)^ As compared to healthy individuals without CHIP, mutational patterns were found to be enriched in SBS4 and SBS6.^(57)^ SBS4 is associated with tobacco smoking, which is also linked to CHIP.^(58)^ On the other hand, SBS6 is associated with defective DNA mismatch repair, raising the possibility that this deficiency is partially responsible for CHIP mutations in some individuals.

Although they also harbor pronounced clock-like mutational signatures, many lymphoid malignancies have signatures that are not typically found in myeloid malignancies. These include those attributed to APOBEC, activation-induced cytidine deaminase activity, DNA polymerase eta, UV light exposure, and MMR deficiency.^(47)^

Recently, Alberge et al.^(59)^ characterized mutational signatures in a cohort of patients with multiple myeloma (*n* = 812) and its precursors: Monoclonal Gammopathy of Undetermined Significance (MGUS; *n* = 37) and smoldering multiple myeloma (*n* = 120). They found 21 different signatures across the cohort, of which eight were novel. Observed signatures with known etiologies included those associated with somatic hypermutation, activation-induced cytidine deaminase (SHM/AID), APOBEC, and ROS, along with the expected clock-like signatures. Clonal hierarchy analysis allowed the authors to determine the temporal order of the different classes of signatures. They concluded that early mutations were largely driven by clock-like signatures and an AID signature before the onset of MGUS and smoldering multiple myeloma. APOBEC-driven mutational processes take hold after the initiation of these precursor conditions and then gradually develop into multiple myeloma.

Similar to solid tumors, patients with hematological malignancies who have undergone treatment typically carry signatures of the therapy. Indeed, analysis reveals signatures specifically attributed to thiopurine chemotherapy,^(36)^ platinum chemotherapy,^(35,47,60)^ melphalan,^(61)^ and/or radiation treatment^(35)^ that the patient had undergone.

Table 2 catalogs the mutational signatures that have been reported to date in hematological malignancies.

**Table 2:** Description of the mutational signatures most commonly found in hematologic malignancies

Mutational process	SBS signatures	Malignancy type
Aging (clock-like signatures)	SBS1 and 5	Ubiquitous signatures
Activity of APOBEC cytidine deaminase	SBS2 and 13	MM, MGUS, SMM, DLBCL, FL, B-ALL
Defective homologous DNA repair	SBS3	MM
Defective DNA base excision repair	SBS30	MM
AID	SBS84 and 85	MM, MGUS, SMM, DLBCL, FL, B-ALL, CLL
Ultraviolet light exposure	SBS7	DLBCL and mature T and NK neoplasms
Polymerase eta somatic hypermutation	SBS9	MM, MGUS, SMM, DLBCL
Defective DNA MMR	SBS6, 15, 20, 21, 26 and 44	MM, DLBCL, FL, BLL, mature T and NK neoplasms
ROS	SBS18	MM, MGUS, SMM, B-ALL, T-ALL and AML
Unknown chemotherapy exposure	SBS86	ALL and B-ALL
Thiopurine chemotherapy exposure	SBS87	ALL and B-ALL
Platinum based chemotherapy exposure	SBS31 and 35	AML and MM
Melphalan exposure	SBS-MM1 (or 99)	AML
Unknown aetiology	SBS8, 16 and 17	MM, MGUS, SMM

AID: activation-induced cytidine deaminase; ALL: acute lymphoblastic leukemia; AML: acute myeloid leukemia; B-ALL: B cell ALL; CLL: chronic lymphocitic leukemia; DLBCL: diffuse large B cell lymphoma; FL: follicular lymphoma; MGUS: monoclonal gamopathy of undetermined significance; MM: multiple myeloma; MMR: mismatch repair; NK: natural killer; SBS: single base substitution; SMM: smoldering multiple myeloma.

## CONCLUSION AND FUTURE WORK

Despite the significant progress made in the study of mutational signatures over the past decade, substantial room remains for further discovery and refinement. One of the main challenges is the variability in the outputs generated by different signature extraction tools, which makes it difficult to determine the most accurate or biologically meaningful results. Currently, most reference signature sets are designed to represent mutational processes across the entire human body. However, it is well established that mutational processes vary significantly across tissues. The development of tissue-specific reference signatures could help address issues such as signature bleeding and improve the biological relevance of signature assignments.

In addition to improving the reference sets, it would be valuable to establish guidelines for tool selection based on cancer type or tissue context, as different tools show varying levels of accuracy depending on the setting. Similarly, a better understanding of the minimum number of mutations required to reliably extract each signature is required. Some signatures, particularly the flatter ones, are inherently more difficult to detect, and applying signature analysis to low-mutation samples without this knowledge may lead to misleading conclusions.

Another promising avenue for future research is the application of mutational signatures to noncancerous tissues. As this methodology expands beyond oncology, it opens up a broad range of opportunities for exploring mutational processes in other biological contexts such as aging, inflammation, and disease precursor states.

Specifically within hematologic malignancies, research on mutational signatures remains relatively underdeveloped, particularly for myeloid neoplasms. Given the rapid evolution of tools and the increasing availability of large-scale public datasets, there is an opportunity to close this gap, even in the face of relatively low mutation rates. Table 3 provides a sample of publicly-available whole-exome and whole-genome data sets from hematological malignancies. Most of these data sets have not been mined for mutational signatures. Applying mutational signature analysis to these diseases may also shed light on pre-leukemic conditions such as clonal hematopoiesis of indeterminate potential and clonal cytopenia of unknown significance, which are well studied in the literature but are not yet fully understood from a mutational process standpoint. Indeed, viewing mature hematological malignancy as the terminus of a continuous evolution from normal hematopoietic stem cells to precursor conditions to frank malignancy, examination of mutational signatures at each of these stages may provide clues as to which signatures are associated with progression. Thus, accurate characterization of mutational signatures in the early stages of leukemia development may aid in clinical diagnosis, prognosis, and treatment decisions.

**Table 3:** Exemplary publicly-available data sources for mutational signature inference

Data source	Data types	Disease subtype(s)	Number of patients
TARGET	WES, WGS	ALL	1006
TARGET	WES, WGS	AML	249
TCGA	WES, WGS	AML	200
TCGA	WES, WGS	Diffuse Large B-cell Lymphoma	58
BeatAML	WES	AML	534
MMRF-CoMMpass	WES, WGS	Multiple Myeloma	975
MP2PRT	WES, WGS	ALL	1507
CGCI	WGS	Burkitt Lymphoma	252
CGCI	WGS	Diffuse Large B-cell Lymphoma	66
The CLL Genome Project	WES, WGS	Chronic Lymphocytic Leukemia	284
MDS Sequencing Project	WES	Myelodysplastic Syndrome	42
All of Us	WGS	AML, MDS	1020
OHSU	WES	Chronic Myeloproliferative Disorders	158

ALL: acute lymphocytic leukemia; AML: acute myeloid leukemia; CGCI: the Cancer Genome Characterization Initiative; CLL: chromic lymphocytic leukemia; MDS: myelodysplastic syndrome; MMRF-CoMMpass: Multiple Myeloma Research Foundation-Relating Clinical Outcomes in Multiple Myeloma to Personal Assessment of Genetic Profile; MP2PRT: Molecular Profiling to Predict Response to Treatment; OHSU: Oregon Health Sciences University; TARGET: Therapeutically Applicable Research to Generate Effective Treatments; TCGA: the Cancer Genome Atlas; WES: whole-exome sequencing; WGS: whole-genome sequencing.

## Data Availability

The underlying content is contained within the manuscript.
